# Consideration of Management Techniques for Advanced Hepatocellular Carcinoma With Metastasis to the Right Atrium: A Case Report

**DOI:** 10.7759/cureus.24614

**Published:** 2022-04-30

**Authors:** Brandon C Warren, Harika Yadav, Nicholas Campbell, Steven Colby, Laura Youngblood

**Affiliations:** 1 Internal Medicine, University of Tennessee, Chattanooga, USA

**Keywords:** angio vac, transcatheter arterial embolization, metastatic liver cancer, right atrial thrombus, hepatocellular carcinoma (hcc)

## Abstract

Hepatocellular carcinoma (HCC) is a common form of cancer and the most common form of liver cancer. Multiple etiological factors leading to HCC include hepatitis B and C, diabetes, alcoholic fatty liver disease, and non-alcoholic fatty liver disease. Hepatocellular carcinoma in the late stages may present with tumor burden and thrombi that can extend into the right atrium (RA). This late-stage form of HCC has a poor prognosis. In this case, we present a 63-year-old male who presented to the hospital with acute encephalopathy with bilateral pulmonary emboli and a thrombus secondary to HCC extending into the RA. Clinical trials for non-surgical interventions are ongoing and are needed to treat patients with tumor burden who may be at bleeding risk from tumor resection.

## Introduction

Hepatocellular carcinoma (HCC) is one of the most common forms of cancer globally, and it is the most common form of liver cancer [1]. Multiple etiologies of HCC exist; however, cirrhosis is the leading risk factor for the development of HCC regardless of the initial cause of cirrhosis which may include causes such as alcoholic cirrhosis, hemochromatosis, Wilson’s disease, and hepatitis B and C [2]. Advanced HCC can rarely lead to tumor burden metastasizing to the inferior vena cava (IVC) with concomitant thrombi extending into the right atrium (RA). This extension of the thrombi with associated tumor burden has a poor prognosis. Traditional non-surgical treatment for a right atrial thrombus includes anticoagulation with heparin bridged to long-term warfarin therapy or thrombolytic therapy. Surgical management remains the mainstay of a multimodal treatment plan involving resection of the mass and partial hepatectomy. Recent advances in immunologic pharmaceutical development are also promising for increasing the overall survival time of patients with advanced HCC.

## Case presentation

**Figure 1:**
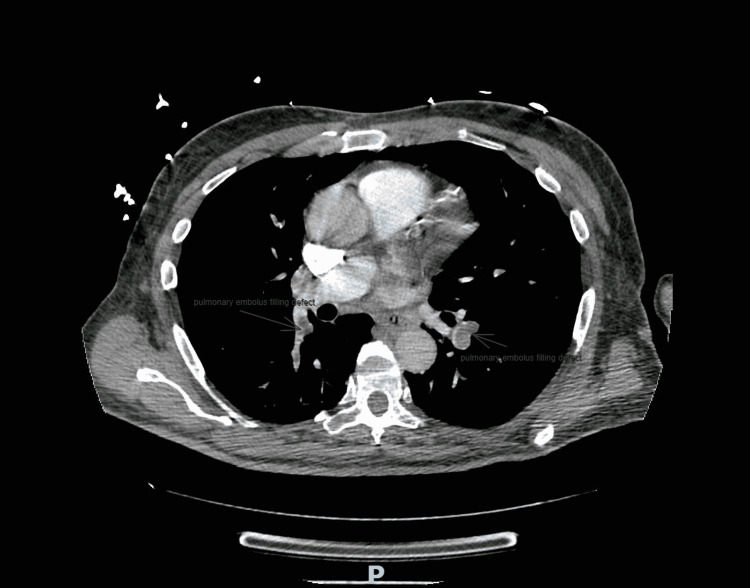
Computerized tomography of the chest showing bilateral pulmonary emboli filling defects.

**Figure 2:**
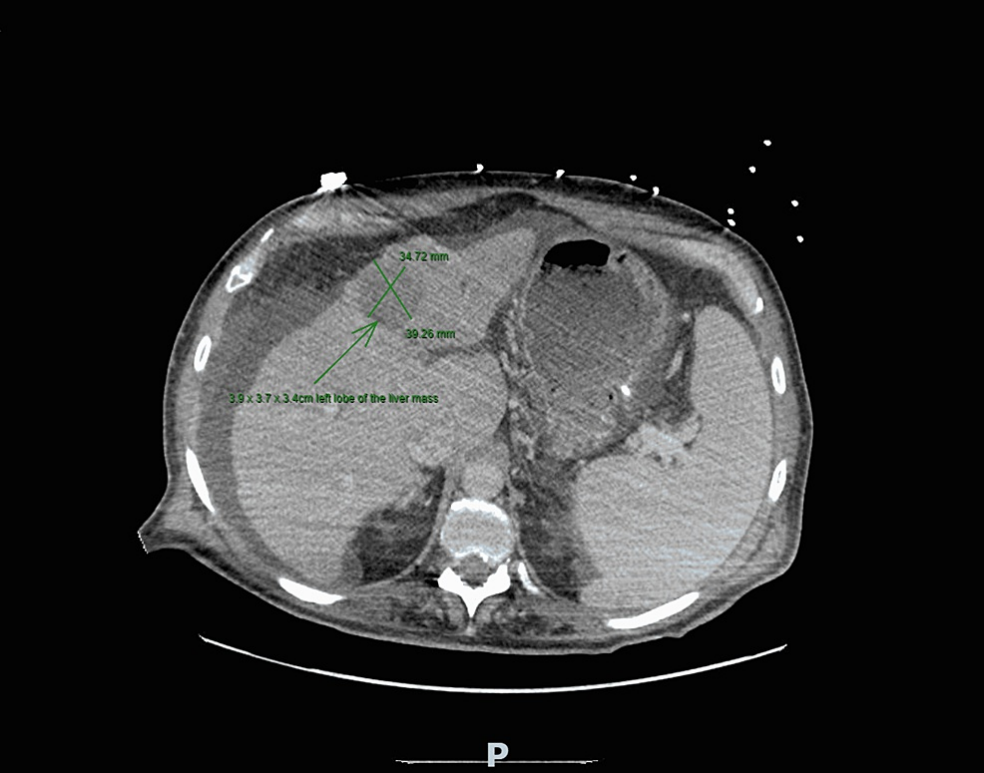
Computerized tomography scan of the abdomen showing a 3.9 × 3.7 × 3.4 cm left lobe of the liver mass.

A 63-year-old male with a medical history of hypothyroidism, type 2 diabetes mellitus, and recent hemorrhagic stroke one month prior to admission presented to the emergency department with acute encephalopathy. Initial computerized tomography (CT) of the head was negative for hemorrhage but incidentally showed a bilateral pulmonary embolism (PE) (Figure 1). A computerized tomography angiography (CTA) chest was ordered which confirmed partial occlusion in the lobar pulmonary arteries, as well as a left lobe of the liver hypodense 3.5 × 2.1 cm lesion, which after further CT abdominal imaging and liver biopsy would reveal HCC (Figure 2).

Due to the acute presentation of the encephalopathy, there was a concern for brain metastases from a previously unknown tumor. A magnetic resonance imaging (MRI) of the brain revealed diffuse embolic infarcts. His echocardiogram on a previous admission noted a patent foramen ovale. A transesophageal echocardiogram (TEE) during the admission was obtained and showed a large heterogeneous mobile structure in his IVC extending into his RA concerning for a thrombus versus a mass.

Despite having a recent hemorrhage, the patient was trialed on anticoagulation to prevent a new stroke or a massive PE. Unfortunately, he did not tolerate this due to a new bleeding 2 × 3 cm right buccal mass not amenable to cauterization. This mass was biopsied and was consistent with HCC. 

His prognosis with metastatic HCC to the mouth, numerous cardioembolic cerebrovascular accidents, bilateral PE, cirrhosis with child-pugh class B, a large IVC, and an RA thrombus/mass with confusion and debility was estimated to be weeks to months without treatment. After many multidisciplinary meetings and discussions with the family, his care was transitioned to a comfort focus. He was discharged to hospice care and died within two weeks.

## Discussion

Thrombi are infrequently found in a structurally normal heart; however, they may be seen in patients who have a hypercoagulable state, inflammatory state, or malignant tumors [3]. In HCC, it is rare to see pulmonary emboli associated with large tumor thrombi as the initial presenting manifestation. Advanced HCC generally presents initially with symptoms of jaundice, pruritus, hepatomegaly, splenomegaly, hepatic encephalopathy, or ascites associated with cirrhosis secondary to the HCC [4]. Even rarer is that in this patient, there were no symptoms related to his PE, including tachycardia, shortness of breath with hypoxia, or hypotension despite a significant clot burden, and his thrombus was found incidentally.

Rarely, patients with HCC may present with a thrombus extending from the IVC into the RA. This is due to invasion of the hepatic vein and IVC by the tumor forming a thrombus that traveled through the IVC up to the RA. This is a rare condition indicating a very poor prognosis. A consensus for the management of patients with advanced HCC with tumor thrombus has not been established. Treatment options include pharmacological (anticoagulation), surgical resection to reduce tumor burden, or palliative options including chemotherapy, radiation, or combination regimens [1,3]. In this patient, AngioVac as a minimally invasive approach was considered. AngioVac allows en-bloc removal of a large amount of thrombus while avoiding thrombolytics in patients with a high risk of bleeding [5], but was not done in this case due to concern for tumor seeding during the procedure.

Management of advanced HCC is an ongoing field of research. Literature review shows that surgical resection of the thrombus with no further surgical resection of the liver confers an average survival time of six months [6]. Further treatment modalities have advanced in recent years including transarterial chemoembolization (TACE) which angiographically visualizes blood vessels involved in the supply of the tumor with embolization of the vessels to help prevent further growth and shrink the tumor [6]. Risks of TACE include further pulmonary emboli formation or ischemic hepatic necrosis. Other treatment modalities include treatment with medications such as Sorafenib which is a protein kinase inhibitor that improves both time to progression and overall survival time of the patient [7]. With the advancement of surgical techniques, including hepatectomy and postoperative management, mean survival has improved to 20 months [3]. Some patients will not benefit from management with surgical intervention due to comorbidities that make surgical intervention risky, and these patients will benefit from further research into non-surgical options for the management of advanced HCC. Further investigation into both surgical and non-surgical management techniques is needed to further increase the time to progression and overall survival time of the disease.

## Conclusions

Advanced HCC with the extension of a thrombus into the RA is a rare presentation. Multiple case reports have been published showing that advanced HCC with extension into the RA has a poor prognosis for patients even after surgical resection of the liver with a median survival time of 20 months. Despite this, clear guidelines for management are not readily available. It is important to note that HCC is a highly vascular tumor, especially when metastasized such that anticoagulation or thrombolytics are contraindicated. We believe it is of increasing importance to develop new therapies for less invasive tumor management modalities when patients are found not to be surgical candidates due to their bleeding risk. Some clinical trials are ongoing and may demonstrate further guidance.
